# Role of schools in disaster risk management: a systematic review

**DOI:** 10.1186/s12873-025-01453-z

**Published:** 2025-12-18

**Authors:** Ameneh Marzban, Mohsen Dowlati, Shandiz Moslehi, Milad Ahmadi Marzaleh

**Affiliations:** 1 Department of Health in Disasters and Emergencies, School of Health Management and Information Sciences, Iran University of Medical Sciences, Tehran, Iran; 2 Health Management and Economics Research Center, Health Management Research Institute, Iran University of Medical Sciences, Tehran, Iran; 3 Department of Health in Disasters and Emergencies, School of Health Management and Information Sciences, Shiraz University of Medical Sciences, Shiraz, Iran

**Keywords:** Disasters, Prevention, Preparedness, Response, Recovery, Schools

## Abstract

**Background:**

Community-based disaster risk management has emerged as a highly effective approach, emphasizing the importance of local institutions. As integral parts of communities, schools possess valuable resources that can play a crucial role in supporting government agencies in managing disasters efficiently. Therefore, this study identified the dimensions and components of schools participation in disaster risk management.

**Methods:**

A comprehensive search was conducted across key electronic databases, including PubMed, Web of Science, and Scopus, focusing on English-language articles published up to June 8, 2024. Additionally, searches were performed on organizational websites such as WHO, CDC, FEMA, IFRC, UN, INEE, and Save the Children. Study selection followed the PRISMA 2020 guidelines, and thematic analysis was employed to examine the findings.

**Results:**

Finally, of 7824 selected records, 17 papers were included in the final analysis. Six main themes, 26 categories, and 61 subcategories were revealed. The main themes included planning and preparedness, education and awareness, Communication and Collaboration, Equipment and Infrastructure, Evaluation and Improvement, Challenges and Solutions.

**Conclusions:**

Schools are central to disaster risk management and must be integrated into national frameworks through formal legislation. Strengthening preparedness requires investment in infrastructure, regular assessments, and inclusive education programs. Stakeholder collaboration especially with families, NGOs, and local authorities enhances coordination and community awareness. Sustainable funding and flexible, localized strategies are essential, particularly in underserved areas. Future research should focus on cost-effective models, digital tools, and scalable practices across diverse contexts.

**Supplementary Information:**

The online version contains supplementary material available at 10.1186/s12873-025-01453-z.

## Introduction

Disasters, both natural and human-made, pose significant threats to communities, often leading to devastating consequences for individuals and infrastructure [1–8]. As societies work towards reducing disaster risks, the importance of community-based disaster risk management (CBDRM) has grown, highlighting the need for local institutions to take an active role in preparedness and response [9–11]. CBDRM refers to a participatory approach that empowers communities to assess risks, plan locally appropriate strategies, and mobilize resources to reduce vulnerability and enhance resilience. This framework provides the theoretical foundation for this study, emphasizing the role of schools as embedded community institutions capable of contributing to all phases of disaster management [12, 13].

Among these institutions, schools stand out as crucial actors in ensuring resilience, preparedness, and effective disaster management [14]. Rather than reiterating their potential repeatedly, this review focuses on the specific and underexplored roles schools can play across the disaster management cycle [15].

Schools are more than just educational spaces; they serve as community hubs where knowledge, communication, and coordinated action can significantly impact disaster response [16, 17]. By integrating disaster management principles into school environments, educational institutions can foster a culture of awareness, preparedness, and proactive response, benefiting both students and the broader community [18].

Educational institutions possess valuable resources, including trained personnel, structured curricula, and established communication networks, all of which can be harnessed to support disaster risk management efforts [19]. Their strategic positioning within communities enables them to act as centers for training, emergency coordination, and recovery support during disaster situations [20]. Schools can contribute across all phases of the disaster management cycle including prevention, preparedness, response, and recovery by offering early education, facilitating drills, coordinating with emergency services, and supporting post-disaster rehabilitation. This multidimensional capacity highlights the importance of schools as operational partners in DRM, especially in resource-limited settings [21].

Despite their potential, schools often remain underutilized in comprehensive disaster risk management strategies [22]. While some schools engage in short-term preparedness activities, such as drills for earthquakes or fire safety, their involvement in broader prevention, response, and recovery efforts is often overlooked [23]. Existing research highlights the necessity of disaster education, emphasizing how early exposure to risk management concepts can significantly improve response outcomes [5]. Students and educators trained in disaster preparedness are not only better equipped to respond in emergencies, but they also serve as valuable resources in spreading awareness within their communities [24]. To enhance contextual relevance, this review incorporates region-specific examples from Asia, Africa, and Latin America, where schools often serve as frontline institutions in disaster response and recovery [15, 16, 25].

Previous reviews have primarily focused on disaster education or school safety protocols. However, they have not systematically synthesized the full range of school participation across all phases of the DRM cycle [21, 22, 26]. This study addresses that gap by identifying and categorizing the dimensions and components of school involvement, offering a more integrated and operational perspective.This study investigates the role of schools in disaster risk management through a systematic review of existing literature. By identifying gaps, synthesizing key themes, and offering evidence-based recommendations, it aims to inform policymakers, educators, and emergency planners on how to better integrate schools into DRM frameworks.

## Methods

This systematic review was conducted in accordance with the PRISMA 2020 guidelines [27] to ensure transparency and reproducibility in reporting. The study protocol was prospectively registered on PROSPERO (ID: CRD42025648766).

### Search strategy

To identify relevant literature, we developed a comprehensive search strategy centered on the intersection of “disaster risk management” and “school participation”. Keywords included “Disasters,” “Disaster Risk Management,” “Schools,” “Educational Institutions,” and “School Participation.” Synonym identification was supported by Medical Subject Headings (MeSH) and prior literature. Searches were conducted across three major databases PubMed, Web of Science, and Scopus. For each database, tailored search syntaxes were applied using Boolean operators and controlled vocabulary where applicable. For example, in PubMed, MeSH terms were combined with title/abstract keywords; in Web of Science and Scopus, keyword combinations were adapted to each platform’s indexing system. The search timeframe extended up to June 8, 2024, without any restriction on the start date, allowing for the inclusion of all relevant literature published in English. Additionally, gray literature and organizational reports were retrieved from websites of WHO, CDC, FEMA, IFRC, UN, INEE, and Save the Children. The detailed PubMed syntax is as follows:

((disaster* [Title/Abstract] OR disasters [MH] OR emergency* [Title/Abstract] OR emergencies [MH] OR hazard* [Title/Abstract] OR “crisis management” [Title/Abstract] OR “emergency management” [Title/Abstract] OR “incident management” [Title/Abstract] OR “accident management” [Title/Abstract] OR “event management” [Title/Abstract] OR “catastrophe management” [Title/Abstract] OR “disaster planning” [Title/Abstract] OR “disaster planning” [MH] OR “disaster risk” [Title/Abstract] OR “risk management” [Title/Abstract] OR “risk management” [MH] OR “disaster risk reduction” [Title/Abstract] OR “relief planning“[Title/Abstract] OR “disaster risk management” [Title/Abstract] OR “disaster management” [Title/Abstract] OR “disaster mitigation” [Title/Abstract] OR “disaster prevention” [Title/Abstract] OR “disaster preparedness” [Title/Abstract] OR “disaster response” [Title/Abstract] OR “disaster recovery” [Title/Abstract])) AND (School* [Title/Abstract] OR Schools [MH] OR “educational organizations” [Title/Abstract] OR “educational institutions” [Title/Abstract] OR “primary school” [Title/Abstract] OR “secondary school” [Title/Abstract] OR “high school” [Title/Abstract] OR student* [Title/Abstract] OR teacher* [Title/Abstract] OR “school managers“[Title/Abstract] AND (“School participation“[Title/Abstract] OR participation [Title/Abstract] OR participatory [Title/Abstract] OR leadership [Title/Abstract] OR leadership [MH] OR partnership[Title/Abstract]))

### Inclusion and exclusion criteria

To ensure methodological transparency and reinforce the credibility of the review, the inclusion and exclusion criteria were developed with careful attention to both relevance and conceptual alignment with the research objectives. We included peer-reviewed studies published in English that employed qualitative, quantitative, or mixed-methods designs and explicitly examined the role of schools in community-level disaster risk management. The decision to include a broad range of empirical designs was intended to capture the diversity of evidence and perspectives represented across the literature, given the multidisciplinary nature of disaster risk management and school participation.

Studies were excluded if full-text access was unavailable, as restricted access would hinder accurate appraisal and data extraction. Publications in languages other than English were excluded due to resource limitations for reliable translation and quality assessment. Additionally, studies were not included if their primary focus did not relate to school-based or community-connected aspects of disaster risk management, ensuring conceptual relevance to the review question. Review articles, commentaries, editorials, and purely theoretical works were excluded to maintain a synthesis grounded in original empirical evidence.

### Screening

**Fig. 1:**
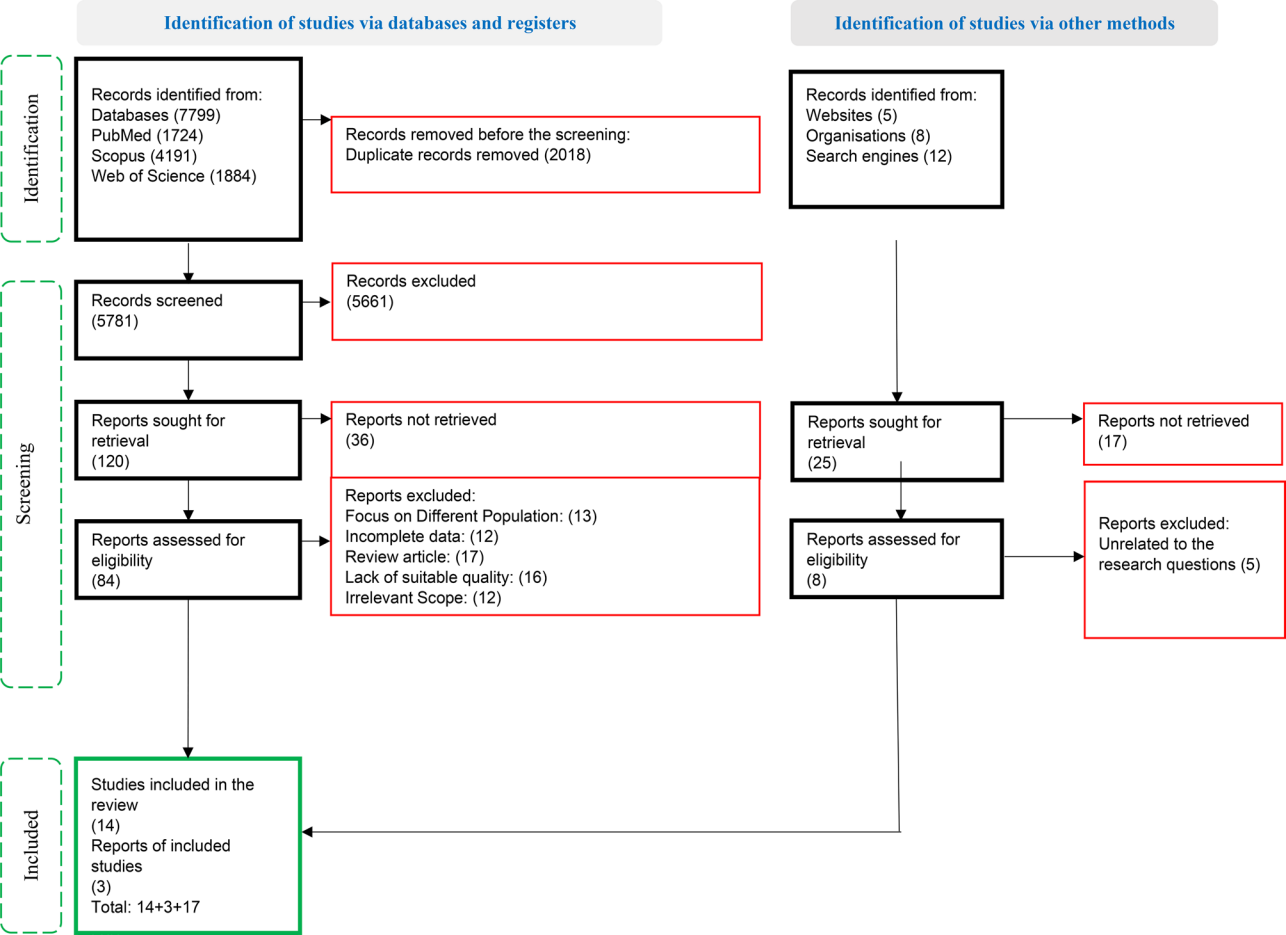
PRISMA2020 flow diagram

Initial search results were deduplicated using EndNote X8 reference management software. Titles and abstracts were screened independently by two reviewers. Full texts of potentially eligible studies were then assessed. Disagreements were resolved through discussion or consultation with a third reviewer. Quality appraisal was conducted using standardized tools, and low-quality studies were excluded. Double assessments were conducted during the quality appraisal phase to ensure reliability and minimize bias. Manual searches supplemented the database results (Fig. 1).

### Data extraction

A structured data extraction form was developed to capture key study characteristics, including author, year, location, methodology, objectives, and findings. Two reviewers independently extracted data, and discrepancies were resolved with input from a third researcher. Extracted data were tabulated for synthesis (see Supplementary Table 1 in Appendix).

### Data analysis

We employed thematic analysis to identify recurring patterns across the selected studies. This approach allowed for systematic coding and theme development, facilitating a nuanced understanding of school involvement in disaster risk management.

### Data analysis procedure

The data analysis was carried out through a six-phase thematic analysis process. These phases included familiarization with the data, generating initial codes, identifying potential themes, reviewing and refining those themes, defining and naming them, and finally producing the report [28]. To extract meaningful codes, the researchers repeatedly reviewed the selected studies, organizing initial codes into subcategories. These subcategories were then grouped into broader categories, which led to the identification of six main themes. The entire analytical process was supported by MaxQDA 2020 software, ensuring systematic coding and organization of data.

### Eligibility and quality appraisal

Following the selection of studies for full-text review, a critical appraisal was conducted to assess methodological quality using the Joanna Briggs Institute (JBI) tool [29]. This tool provides tailored checklists for various study designs; accordingly, we applied the relevant checklists for qualitative research, cross-sectional studies, textual sources (including books, reports, guidelines, and other documented materials), and gray literature. Each checklist item was scored as follows: 1 point for “Yes” (criterion met), 0 points for “No” (criterion not met), and 0.5 points for “Unclear” (criterion uncertain). Final scores were calculated for each study, and to enhance transparency in quality assessment, studies were categorized based on their total scores: 0–3 indicating weak quality, 4–5 moderate quality, and 6–8 strong quality. Results from the risk-of-bias assessment indicated that over 60% of the included studies were rated as strong, with no studies classified as weak.

### Data synthesis and thematic analysis

The results were synthesized using thematic analysis to identify recurring patterns and concepts related to school participation in disaster risk management. Themes were derived inductively and refined through iterative coding. This approach allowed for a structured interpretation of findings across diverse study designs.

## Results

A total of 7824 articles were identified through database searches, Websites, Organisations and Search engines. After eliminating 2018 duplicate entries, the titles and abstracts of the remaining studies were reviewed, leading to the exclusion of 5,661 studies. Additionally, 36 studies could not be retrieved. As a result, 84 studies were selected through screening, along with 8 studies identified through manual search, both of which were reviewed by two independent reviewers. Ultimately, 17 studies proceeded to the data extraction phase. After analyzing the data, six main themes, 27 categories, and 61 subcategories were extracted (Table 1).

### Descriptive statistics

**Fig. 2:**
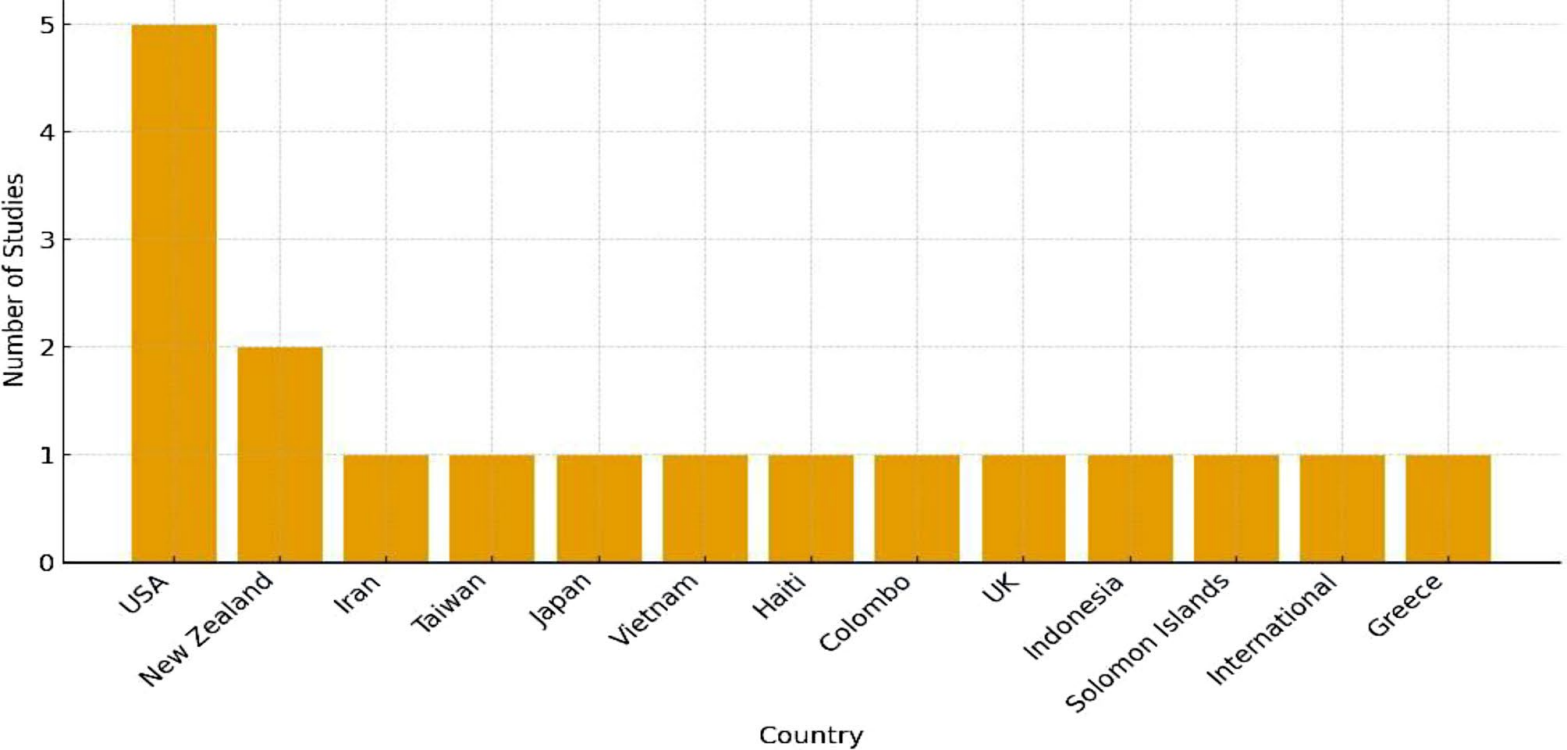
Methodological distribution of included studies

A total of 17 studies were selected, spanning publication years from 2005 to 2023. Geographically, the studies were predominantly conducted in high-income countries, especially the United States (5 studies), with limited representation from low-income regions. The study settings included the United States (5 studies), New Zealand (2 studies), Iran (1 study), Taiwan (1 study), Japan (1 study), Vietnam (1 study), Haiti (1 study), Colombo (1 study), the United Kingdom (1 study), Indonesia (1 study), Solomon Islands (1 study), an international study (1 study), and Greece (1 study) (Fig. 2).

**Fig. 3:**
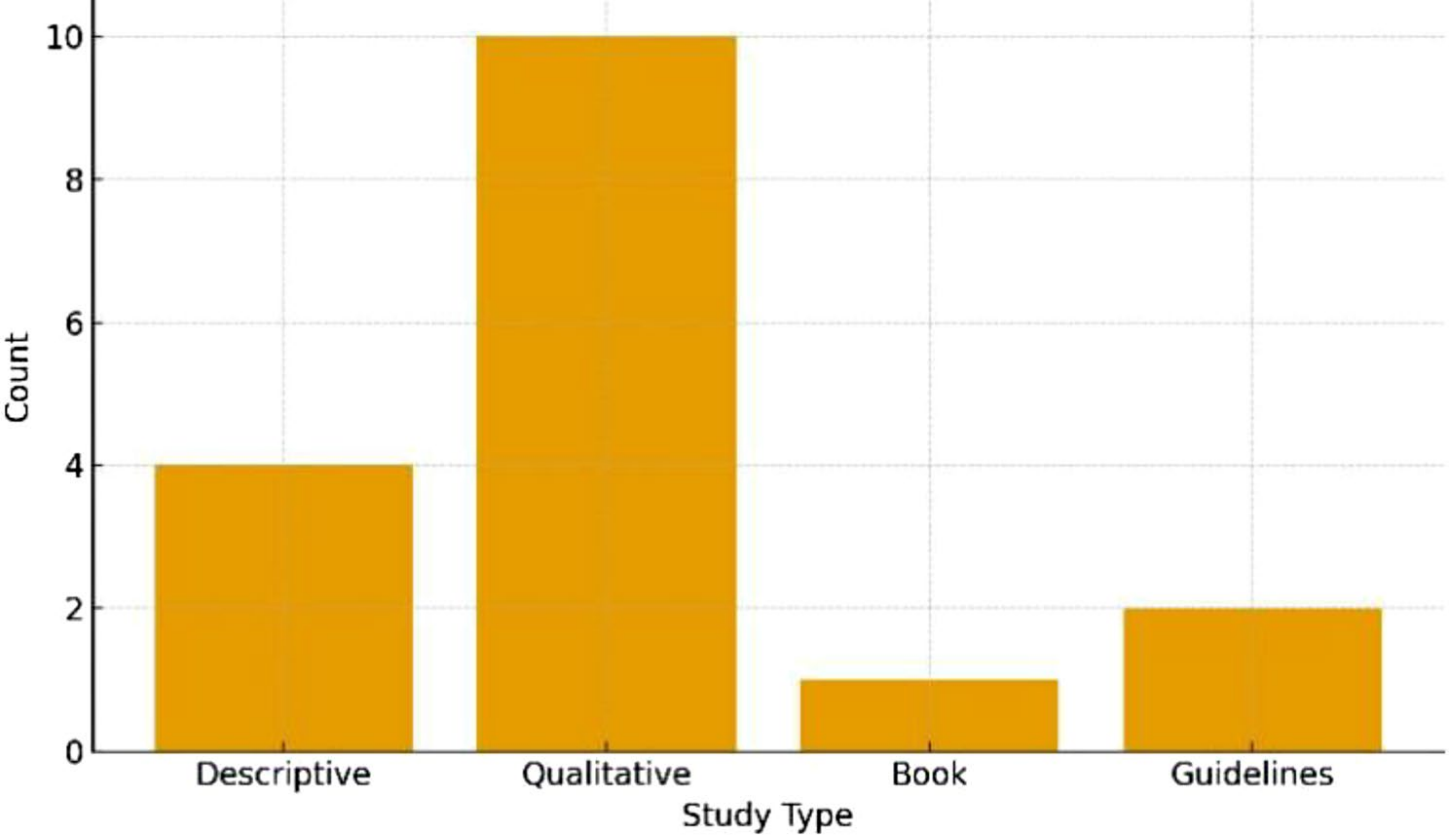
Geographical distribution of included studies

In terms of methodology, the selected studies comprised 4 descriptive studies, 10 qualitative studies, 1 book, and 2 guidelines. Based on the quality assessment, 12 studies were classified as strong, while 5 studies demonstrated moderate quality (Fig. 3).

**Table 1:** Dimensions and components of school participation in disaster risk management

Theme	Category	Subcategory
Planning and preparedness	Developing school disaster management programs	Identifying potential threats in schools
		Formulating response strategies for schools
		Creative approaches by school principals to handle crises
		Evaluating Needs and Available Resources
		Creating and Distributing Guidelines in Schools
	Establishing disaster committees in schools	Defining roles and responsibilities of disaster committee members
		Conducting regular meetings of the disaster committee
	Preventive actions for disasters in schools	Identifying natural and human-made risks
		Assessing and evaluating local risks
	Psychological and social preparedness for students, staff, and parents	Providing psychological services and counseling in schools
		Implementing special educational strategies and social support networks
Education and awareness	Student training on disaster risks	Educational and study programs in schools
		Conducting practical drills for students
	Workshops and training courses for teachers and staff	Discussions on safety norms
		Methods of communicating information to students
	Community awareness	Hosting briefing sessions for parents
		Distributing educational resources for families
		Organizing seminars and awareness meetings for parents and the local community
Communication and collaboration	Engagement with NGOs and families	Identifying relevant organizations
		Establishing collaboration agreements
	Building cooperation networks between schools and local institutions	Holding joint meetings
		Utilizing local expertise
	Sharing experiences with other schools in disaster management	Organizing conferences and seminars
		Creating online support groups
	Interaction and cooperation with government authorities	Establishing connections with education departments
		Collaborating in the development of local policies
		Coordinating joint drills with government entities to enhance preparedness
		Exchanging information and experiences
Equipment and infrastructure	Providing essential equipment for disaster management in schools	Stocking emergency kits in schools
		Installing alarm systems for early warnings
		Maintaining and ensuring preparedness of school equipment
	Enhancing school physical infrastructure (shelters, safety zones)	Designing and arranging secure spaces within schools
		Developing safety maps for schools
		Assessing the need for maintenance of school facilities
	Developing rescue and evacuation systems in schools	Creating structured evacuation procedures
		Conducting frequent evacuation drills in schools
	Assessing and improving physical safety in schools	Regularly evaluating structural conditions
		Strengthening school buildings against disasters
		Implementing safety enhancement programs
	Role of school nurses	Acting as liaisons between students and healthcare services
		Identifying and tracking close contacts in health-related situations
Evaluation and improvement	Continuous assessment of school disaster management programs	Utilizing evaluation methodologies
		Preparing periodic reports
	Identifying strengths and weaknesses in disaster response	Conducting trial-and-error scenarios
		Analyzing feedback
	Gathering feedback from students and families	Creating surveys and questionnaires
		Organizing feedback sessions
	Updating and refining programs based on experience	Reviewing existing policies
		Adapting to new conditions
Challenges and solutions	Financial challenges in schools	Lack of financial resources for implementing disaster management programs
		Need for funding to repair and rebuild school infrastructure
	Financial solutions for schools	Allocating appropriate funding to school disaster management programs
		Securing financial support from governmental and non-governmental organizations
	Logistical challenges in schools	Difficulties in acquiring essential equipment
		Challenges in distributing necessary materials to schools
		Ensuring access to resources during crises
	Logistical solutions for schools	Detailed planning and coordination with various institutions
	Communication and legal challenges in schools	Deficiencies in communication and information systems within schools
		Lack of adequate regulations to support disaster management programs
	Communication and legal solutions for schools	Improving communication and information systems in schools
		Developing and approving appropriate regulations to support disaster management programs

Analysis of the selected studies revealed that the components of school participation in disaster risk management include the following Planning and Preparedness, Education and Awareness, Communication and Collaboration, Equipment and Infrastructure, Evaluation and Improvement, Challenges and Solutions. The results are described below.

### Planning and Preparedness

The theme of Planning and Preparedness emphasizes the importance of structured, proactive strategies in schools to ensure safety during disasters. Schools begin by identifying specific threats such as natural hazards, security risks, and infrastructure weaknesses and develop tailored disaster management programs. These include evacuation plans, communication protocols, and emergency drills. School principals play a key role by introducing innovative approaches like technology integration and student-led initiatives. Clear and accessible preparedness guidelines, distributed through various platforms, help standardize emergency responses and improve awareness. Regular updates and assessments ensure these plans remain effective in changing risk environments [5–7, 30–32].

In addition to planning, schools enhance preparedness through organizational structures and preventive actions. Establishing disaster committees with defined roles improves coordination and enables timely decision-making during crises. These committees also facilitate collaboration with local authorities and emergency services. Preventive measures such as risk assessments, safety drills, and policy integration help schools address vulnerabilities before disasters occur. Moreover, psychological and social preparedness is recognized as a vital component, with schools offering counseling and support networks to help students, staff, and families cope with trauma and build resilience. Integrating mental health into disaster plans strengthens recovery and promotes long-term well-being [1, 4, 6, 7, 23, 25, 30–37].

### Education and awareness

The theme of Education and Awareness highlights the critical role of inclusive training and outreach in strengthening school resilience. Integrating disaster risk education into the student curriculum ensures that learners understand potential hazards and appropriate response strategies. Practical drills and real-world simulations, often conducted in collaboration with emergency services, improve students’ preparedness and confidence during crises. Continuous evaluation and adaptation of these programs help maintain their relevance and effectiveness. For teachers and staff, workshops and training courses focus on safety norms, communication techniques, and interactive learning methods such as role-playing and simulations. Ongoing professional development ensures that educators stay informed about best practices in disaster management [1, 5, 7, 23, 33, 35, 36]. Beyond the school environment, community awareness is essential for building a cohesive and prepared response network. Schools that host briefing sessions for parents and distribute educational materials empower families to understand their roles during emergencies. Organizing seminars and collaborative meetings with local stakeholders fosters trust and shared responsibility. These efforts not only strengthen the relationship between schools and their communities but also enhance overall disaster preparedness. Sustained engagement and education across all levels students, staff, and families create a culture of safety and proactive risk mitigation [1, 2, 4–6, 30–32, 34, 37].

### Communication and collaboration

This theme highlights the essential role of effective communication and multi-level collaboration in enhancing disaster preparedness in schools. Active engagement with NGOs and families provides schools with access to specialized training, expert support, and long-term resources. Formal collaboration agreements and continuous cooperation with these stakeholders ensure coordinated emergency responses and foster sustainable support systems. Involving families in preparedness initiatives also strengthens community resilience and promotes shared responsibility in crisis situations [4, 5, 7, 23, 25, 33].

Additionally, building strong networks with local institutions and government authorities significantly improves coordination and response capacity. Joint meetings with local agencies and other schools enable the exchange of best practices, risk assessments, and innovative solutions. Participation in conferences, seminars, and online platforms supports continuous learning and real-time collaboration. Furthermore, aligning school strategies with national policies through cooperation with education departments and emergency services ensures consistency, access to resources, and effective implementation of drills. These collaborative efforts collectively enhance institutional resilience and long-term disaster management effectiveness [5–7, 23, 25, 30–38].

### Equipment and infrastructure

Ensuring physical preparedness is a cornerstone of school disaster management. Equipping schools with emergency kits, alarm systems, and communication tools enables timely and effective responses during crises. Regular maintenance and investment in these resources ensure operational reliability. Enhancing physical infrastructure such as designated shelters, safety zones, and evacuation maps provides secure spaces and clear guidance for students and staff. Routine assessments and structural upgrades, including retrofitting, help mitigate risks and reinforce school buildings against potential hazards [1, 2, 4–7, 25, 30–32, 34, 36–38].

In addition to infrastructure, organized evacuation systems and health support play a vital role in preparedness. Schools that develop structured evacuation procedures and conduct frequent drills improve response efficiency and student confidence. Collaboration with emergency services helps refine these systems and align them with local protocols. School nurses contribute significantly by offering medical support, monitoring health risks, and coordinating with healthcare providers. Their role strengthens both immediate response and long-term well-being. Together, these measures build a resilient foundation for managing disasters in educational settings [4, 5, 7, 23, 25, 30–38].

### Evaluation and improvement

In the area of equipment and infrastructure, schools enhance their emergency readiness by providing essential resources such as emergency kits, alarm systems, and communication tools. Regular maintenance and continuous investment in disaster management infrastructure ensure operational reliability during crises. Designing shelters, safety zones, and evacuation maps within school premises helps guide students and staff effectively in emergencies, reinforcing physical safety and preparedness [5–7, 25, 30–33, 35–38].

Additionally, developing structured evacuation systems and conducting regular drills improve response coordination and build confidence among students and staff. Collaboration with emergency services further strengthens these systems. Assessing and upgrading school buildings through retrofitting and safety programs reduces vulnerabilities and boosts resilience. School nurses also play a vital role by offering medical support, monitoring health risks, and coordinating with healthcare providers. Together, these efforts create a comprehensive foundation for effective disaster management in educational settings [2, 4, 5, 7, 23, 30–33, 35, 37].

### Challenges and solutions

Schools face several challenges in implementing effective disaster management programs, particularly financial, logistical, and regulatory barriers. Limited funding restricts their ability to invest in preparedness initiatives, infrastructure upgrades, and emergency training. Logistical issues, such as difficulties in acquiring and distributing essential equipment, further hinder timely and coordinated responses. Additionally, inadequate communication systems and the absence of clear legal frameworks reduce schools’ capacity to manage crises effectively. These challenges collectively weaken institutional resilience and delay critical interventions during emergencies [6, 7, 23, 25, 30, 31, 33, 35–38].

To overcome these obstacles, schools must adopt sustainable solutions through strategic planning and collaboration. Securing financial support from government bodies, NGOs, and community partners enables investment in safety infrastructure and training. Establishing structured supply chains and strengthening partnerships with local authorities improve logistical efficiency and resource accessibility. Enhancing communication systems and advocating for comprehensive legal policies provide schools with the tools and authority needed to implement robust disaster management strategies. Continuous evaluation and adaptation of these solutions ensure long-term preparedness and resilience across educational institutions [2, 4–7, 23, 25, 32–35, 37].

## Discussion

Disaster preparedness in schools is a crucial component of ensuring student and staff safety. A comparative analysis of supporting and opposing studies provides insights into the effectiveness of various preparedness strategies. This section is organized around six core themes derived from the results: planning and preparedness, collaboration and partnerships, infrastructure and equipment, education and awareness, evaluation and continuous improvement, and challenges and solutions. This discussion critically examines the alignment and divergence of findings with existing literature, identifying strengths, gaps, and areas for improvement.

The findings confirm that structured disaster planning, including evacuation drills and curriculum integration, significantly enhances school readiness. These results are consistent with previous studies emphasizing the importance of embedding preparedness into school policies and routines [26, 39, 40]. However, some scholars argue that rigid frameworks may limit adaptability, especially in diverse geographical contexts [41]. A balanced approach that combines standardized procedures with context-sensitive flexibility is therefore essential.

Disaster-related education including training for students, teachers, and families is one of the most effective and low-cost strategies for enhancing school resilience. The findings of this study show that participatory learning programs, peer-based initiatives, and practical drills significantly improve emergency response and reduce psychological and behavioral vulnerability during crises [26, 39–41]. Unlike costly infrastructure upgrades, these educational interventions can be implemented with limited resources and remain highly effective even in under-resourced schools.

However, some studies caution that theoretical education alone, without practical implementation and ongoing evaluation, may not lead to genuine preparedness [24, 26]. To maximize impact, schools should combine educational efforts with field exercises, regular assessments, and active stakeholder engagement. This approach is not only cost-effective but also offers a scalable and sustainable solution for schools facing financial constraints at national and regional levels.

Collaboration with NGOs, families, local institutions, and government entities plays a vital role in disaster preparedness. Studies show that such partnerships provide access to specialized training, shared resources, and policy support [26, 41, 42]. However, over-reliance on external actors may create disparities, particularly in under-resourced schools [39]. Some researchers advocate for self-sustaining models that empower schools to develop internal capacities while maintaining strategic external collaborations.

Physical infrastructure, including shelters, alarm systems, and emergency kits, is foundational to school safety. Research supports the effectiveness of well-maintained facilities and equipment in minimizing disruption and improving emergency response [39, 43]. Nonetheless, some studies question the cost-effectiveness of extensive investments, especially in low-income settings [3]. Community-based resource sharing and prioritization of high-impact interventions are proposed as more sustainable alternatives.

Systematic evaluation and feedback mechanisms are crucial for refining disaster management strategies. Schools that conduct regular assessments and integrate lessons learned from past incidents demonstrate greater adaptability [9, 26, 39]. However, excessive evaluation may lead to administrative overload. Simplified, action-oriented frameworks are recommended to ensure that assessments lead to practical improvements without compromising implementation.

Financial, logistical, and regulatory challenges remain significant barriers to effective disaster preparedness. Limited funding restricts infrastructure development and training efforts [26, 44]. While external support is helpful, efficient resource allocation and cost-effective planning are critical. In addition, weak communication systems and unclear legal frameworks hinder coordination. Strengthening policy infrastructure and ensuring alignment with national safety standards are essential for sustainable preparedness.

### Limitations

This review exclusively considered studies published in English, which may restrict the generalizability of the findings. Furthermore, since many studies were conducted within specific geographic regions primarily the United States the results may not be universally applicable to all contexts or populations.

### Strengths

The analysis integrates a wide variety of study types, including qualitative research, descriptive studies, guidelines, and books, providing a well-rounded perspective on the subject. Additionally, incorporating multiple reviewers for data extraction and quality assessment helped reduce bias and strengthen the reliability of the conclusions.

### Research gaps and future directions

Research on the role of schools in disaster risk management has advanced significantly, yet several critical gaps remain. There is a need for longitudinal studies that evaluate the sustained impact of preparedness interventions over time, particularly in low-resource and high-risk settings. Additionally, limited attention has been given to the integration of digital technologies and remote learning tools in disaster preparedness strategies. The perspectives of students, parents, and marginalized communities are also underrepresented, underscoring the importance of inclusive and participatory research approaches. Future studies should explore scalable, cost-effective models tailored to diverse geographic and socioeconomic contexts, and examine how policy frameworks can better support school-based disaster resilience. Addressing these gaps will enhance the evidence base and inform more adaptive, equitable, and sustainable preparedness strategies.

## Conclusions

Schools play a pivotal role in disaster risk management by implementing structured and comprehensive strategies. Addressing financial, logistical, communication, and legal challenges requires ongoing evaluation and targeted improvement. Through active engagement with NGOs, families, government authorities, and local institutions, schools can significantly enhance their preparedness and response capacities.

The effectiveness of disaster management programs depends on regular assessments, identification of strengths and weaknesses, and integration of feedback into evolving strategies. Investments in essential equipment, infrastructure upgrades, and evacuation systems are critical for ensuring safety during emergencies. Moreover, psychological and social preparedness programs support students, staff, and families in coping with post-disaster impacts.

To improve applicability, stronger policy guidance is recommended. Schools should be formally included in national disaster risk management frameworks through legislation or mandates from Ministries of Education. This institutional recognition would ensure consistent support, accountability, and integration across educational systems.

Looking ahead, future studies should explore the long-term impact of participatory disaster education, cost-effective preparedness models in low-resource settings, and the role of digital technologies in enhancing school resilience. Comparative research across diverse geographic and socioeconomic contexts would also help identify scalable best practices.

To enhance school resilience against disasters, stakeholders should prioritize the development of binding policies such as formally integrating schools into national disaster risk management frameworks through legislation or mandates from Ministries of Education. Establishing sustainable funding mechanisms is also essential, particularly for underserved regions, to support educational programs, safety equipment, and infrastructure improvements. Designing localized and flexible preparedness frameworks that align with national policies while addressing specific community risks can significantly improve effectiveness. Active participation from families, NGOs, and local institutions in planning and implementing preparedness measures plays a vital role in strengthening coordination and raising public awareness. Combined with regular evaluations and psychosocial training, these efforts create a safer and more resilient educational environment.

## Supplementary Information

Below is the link to the electronic supplementary material.

Supplementary Material 1

## Data Availability

The data used to support the fndings of this study are available from the corresponding author upon email request.

## Abbreviations

UN: United Nation
INEE: Inter-agency Network for Education in Emergencies
WHO: World Health Organization
CDC: Centers for Disease Control and Prevention
FEMA: Federal Emergency Management Agency
IFRC: International Federation of Red Cross and Red Crescent
MeSH: Medical Subject Headings
PRISMA: Systematic Reviews and Meta-analyses
