# Supplementary material for: Role of schools in disaster risk management: a systematic review
Source: BMC Emerg Med. 2025 Dec 18;26:23. doi: 10.1186/s12873-025-01453-z (PMC12822327; doi:10.1186/s12873-025-01453-z)
Supplement: Supplementary file 1 — Supplementary Material 1 [file 12873_2025_1453_MOESM1_ESM.docx]

Supplementary Material

Appendix

**Supplementary Table 1. Main information of included studies**

| **No.** | **Author(s)**  **Year** | **Country** | **Quality assessment** | **Methodology** | **Main aim** | **Key findings** |
| --- | --- | --- | --- | --- | --- | --- |
| 1 | **Clettenberg S, et al**  **2011**  **(4)** | **United States** | **Strong** | **Descriptive** | Examining Schools' Response to the Impacts of Natural Disasters, Particularly Hurricanes Katrina and Rita. | -Natural disasters caused psychological distress in students and families, including depression and anxiety.  -Schools played a crucial role in providing social support, psychological services, and counseling.  -Effective strategies included specialized educational programs and the establishment of social support networks.  -Teacher training was essential for identifying and managing students’ psychological issues.  -Collaboration between schools and families was necessary to create a supportive environment for recovery. |
| 2 | **Mutch C,**  **2015**  **(35)** | **United States and United Kingdom** | **Moderate** | **Qualitative** | Examining Factors That Influence School Principals' Behavior During Crises. | -Dispositional factors play a significant role in school principals' decision-making.  -Relational dynamics influence crisis management and strengthen effective communication.  -Contextual social and cultural factors within the school environment affect principals' behavior during crises.  -Principals' approach to crises is shaped by past experiences and their capabilities.  -Support from the school community and other stakeholders contributes to stability.  -Flexibility and adaptability are key to successful crisis management.  -Findings from this study can help improve educational leadership in crisis situations. |
| 3 | **Mutch C,**  **2014**  **(36)** | **New Zealand** | **Strong** | **Qualitative** | The Role of Schools in Disaster Situations, Particularly the 2010–2011 New Zealand Earthquakes. | Schools can serve as centers for social support and education. The importance of pre-crisis planning and preparedness to ensure student safety has been emphasized. Additionally, collaboration between schools, the government, and local communities plays a key role in facilitating recovery and disaster response. The need for specialized training for teachers and school staff in disaster management has been highlighted. Finally, creating safe and resilient learning environments during crises is crucial. |
| 4 | **Codreanu TA, et al**  **2016**  **(5)** | **International** | **Strong** | **Descriptive** | Examining the Need for Collaboration Between Families and Charitable Organizations in Teaching Disaster Risk Management to High School Seniors. | Collaboration between families and charitable organizations is essential for increasing students' awareness. The involvement of both groups can enhance educational outcomes and better prepare students. Additionally, international surveys have shown that students require more practical exercises and educational training in this field. Having a clear and structured educational program, along with family support, are key factors for success. Finally, the article emphasizes the importance of raising public awareness and establishing social support systems. |
| 5 | **Cornelli Sanderson R, et al**  **2016**  **(6)** | **Haiti** | **Moderate** | **Qualitative** | Examining and Strengthening Resilience in Children and Communities Following the 2010 Haiti Earthquake. | There is an emphasis on the importance of social support and coping skill education for children. Additionally, the role of local institutions in improving the situation of children and families has been evaluated. Intervention programs, including recreational and educational activities, have been analyzed to reduce stress. The results indicate that creating supportive environments can accelerate the recovery process. Finally, the study highlights the significance of investing in local resources and strengthening communities after natural disasters. |
| 6 | **Thi T, Shaw R**  **2016**  **(31)** | **Vietnam** | **Strong** | **Descriptive** | Developing and Improving Disaster Risk Reduction Education in Primary Schools. | The main goal of this program is to enhance students' awareness of natural disasters and their preparedness for such events. The results indicate that the training has contributed to increasing children's knowledge and skills. Collaboration between schools and local communities has been effective in the program's success, but challenges such as resource shortages and the need for teacher training remain. Ultimately, this program has helped foster a spirit of cooperation and responsibility among students. |
| 7 | **Mutch C**  **2021**  **(25)** | **New Zealand** | **Strong** | **Qualitative** | Examining the Role of Schools in Assisting Communities in Earthquake Disaster Response. | Earthquakes had a significant impact on education and society. Schools acted as social support centers, providing necessary resources for families. Educators also focused on psychology and emotional assistance for students. Ultimately, the article emphasizes the need for comprehensive planning to ensure preparedness and effective crisis response. |
| 8 | **Flaherty EA,**  **2020**  **(7)** | **United States** | **Moderate** | **Qualitative** | Examining the Role of School Nurses as Researchers and Contact Tracing Observers for COVID-19 Patients. | This study shows that school nurses can serve as a bridge between students and healthcare services. Additionally, their ability to identify and track close contacts can help reduce the spread of the virus. The findings indicate that this integration can improve public health and reduce the burden on the healthcare system. |
| 9 | **Hosseini KA, et al**  **2020**  **(33)** | **Iran** | **Strong** | **Qualitative** | Analysis of Ongoing Efforts to Promote Community-Based Disaster Risk Management in Iran. | The main objective is to enhance community-based disaster risk management. Research indicates that education and awareness can strengthen earthquake preparedness. Additionally, collaboration between schools, families, and local institutions is essential for positively impacting communities. The article emphasizes the importance of cultural development and improving local infrastructure. |
| 10 | **Ramos-Pla A, et al**  **2021**  **(37)** | **United States** | **Strong** | **Qualitative** | Examining School Principals' Leadership and Management During the COVID-19 Crisis. | The article examines how school principals faced challenges during the COVID-19 crisis. They made swift and effective decisions to ensure the health and safety of students and staff. Additionally, they emphasized the importance of clear communication and solidarity within the school community. Principals adapted educational programs and leveraged technology for remote learning, introducing creative approaches to overcome difficulties. The findings indicate that effective leadership can enhance schools' resilience during crises. |
| 11 | **Pratiwi PH, et al**  **2023**  **(38)** | **Indonesia** | **Strong** | **Qualitative** | Integrated Natural Disaster Risk Management in the Educational Process in Schools. | The results indicate that educating students about risks and disaster preparedness not only increases their knowledge but also improves their safety behaviors in daily life. Additionally, collaboration with local communities and relevant institutions has a positive impact on the effective implementation of these programs. Ultimately, improving infrastructure and educational resources in this field is key to successfully reducing community vulnerability. |
| 12 | **Wang J-j,**  **2016**  **(32)** | **Taiwan** | **Strong** | **Qualitative** | Examining the Framework and Practices of Crisis Management in Educational Environments. | This article examines school-based crisis management and emphasizes the importance of preparing students and school staff for natural disasters. Key factors include regular training, the establishment of response teams, and collaboration with local communities. Additionally, the role of technology and information in improving crisis management processes is discussed. The article also analyzes the challenges in implementing crisis management programs. Ultimately, it provides recommendations for enhancing crisis management strategies in schools. |
| 13 | **Kalogiannidis S, et al**  **2022**  **(23)** | **Greece** | **Strong** | **Qualitative** | Examining the Role of Educational Systems as a Hub for Risk and Natural Disaster Management. | The article examines school systems in Greece as centers for risk and natural disaster management, highlighting how schools can play a key role in raising awareness and providing education on hazard management. Research indicates that integrating disaster-related educational programs into school curricula can enhance students' preparedness. Additionally, fostering local and interorganizational collaborations is recommended to strengthen crisis response. The findings emphasize the necessity of community and family involvement in the risk management process. Ultimately, the article underscores the importance of investing in educational infrastructure for sustainable and resilient development. |
| 14 | **Yamato Y, et al**  **2019**  **(34)** | **Japan** | **Strong** | **Descriptive** | Step-Based Spatial Planning and Management for Evacuation Shelters Utilizing Elementary Schools in Japan. | This article examines spatial planning and management for emergency shelters in Japan, utilizing elementary schools as designated sites. The findings indicate that schools can serve as effective refuge centers during natural disasters. Additionally, various crisis management and evacuation processes are analyzed, emphasizing the need for a comprehensive plan to optimize existing resources. This research presents strategies for enhancing preparedness and community cooperation during crises. |
| 15 | **Ronan KR, et al**  **2005**  **(30)** | **United States** | **Strong** | **Book** | Promoting Community Resilience Against Natural Disasters Through the Engagement of Schools, Youth, and Families. | The key findings highlight the importance of preparedness education for understanding social and environmental disasters. Schools, as community centers, can provide educational programs and emotional support. Young people, through active participation in social activities, can have a positive impact on society. Additionally, collaboration between families and educational institutions is essential for strengthening social networks and supporting one another. |
| 16 | **Ministry of Education and Human Resource Development , Solomon Islands , 2011**  **(1)** | **Solomon Islands** | **Moderate** | **Guideline** | Planning for Crisis Management in Schools. | -Threat Identification: Analyzing and recognizing various natural and human-made risks that may impact schools.  -Strategy Development: Creating preventive programs and response measures to reduce potential damages.  - Education & Awareness: Organizing training sessions for teachers and students on crisis management.  -Curriculum Planning: Integrating disaster management topics into school curricula.  -Stakeholder Collaboration: Strengthening connections with local and governmental organizations for crisis support. |
| 17 | **Ministry of Education for Social Cohesion Programme (ESC Programme), Colombo 2008**  **(2)** | **Colombo** | **Moderate** | **Guideline** | This guideline has been developed with the aim of enhancing school safety against natural disasters. | The importance of educating students and school staff on preparedness and crisis management is emphasized.  -Assessing local risks and prioritizing planning for effective responses.  -Collaborating with local institutions and the government to establish resilient infrastructure is essential.  -Emphasis is placed on implementing continuous and updated educational programs to enhance school resilience |
